# Supplementary material for: Preoperative risk assessment and spirometry is a cost-effective strategy to reduce post-operative complications and mortality in Mexico
Source: PLoS One. 2022 Jul 27;17(7):e0271953. doi: 10.1371/journal.pone.0271953 (PMC9328537; doi:10.1371/journal.pone.0271953)
Supplement: Attachment — Submitted filename: Response to revierew comments PONE-D-21-36131 June 9 22.docx [file pone.0271953.s002.docx]

**RESPONSE TO PEER-REVIEW REPORT**

**Name of journal:** PLOS ONE

**Manuscript ID:** PONE-D-21-36131

**Title:** Preoperative risk assessment and spirometry is a cost-effective strategy to reduce post-operative complications and mortality in Mexico

**Corresponding author:** Dr. Yvonne N. Flores

We appreciate all the reviewers ‘comments, which have helped to improve our manuscript. The modified lines of text we refer to in our response are from the revised version of our manuscript with track-changes.

**RESPONSE TO SPECIFIC COMMENTS:**

**To the Editor:**

1. In ethics statement in the manuscript and in the online submission form, please provide additional information about the patient records/samples used in your retrospective study. Specifically, please ensure that you have discussed whether all data/samples were fully anonymized before you accessed them and/or whether the IRB or ethics committee waived the requirement for informed consent. If patients provided informed written consent to have data/samples from their medical records used in research, please include this information.

**Response 1:** As suggested by the Editor, we have added the following information to the Methods section: “Patient informed consent was not required because the HGM ethics committee waived the requirement for informed consent. The HGM authorized YMG to extract the information from the patient charts that was used for this analysis. All data were fully anonymized using a unique code to identify each patient. The analyzed data did not contain any information that could reveal the identity of the patients [28].” Page 8, lines 128-132 (clean file).

2. We note that you have provided additional information within the Acknowledgements Section that is not currently declared in your Funding Statement. Please note that funding information should not appear in the Acknowledgments section or other areas of your manuscript. We will only publish funding information present in the Funding Statement section of the online submission form.
   Please remove any funding-related text from the manuscript and let us know how you would like to update your Funding Statement. Currently, your Funding Statement reads as follows:

The author(s) received no specific funding for this work.

**Response:** Thank you for the opportunity to clarify this issue. As we mentioned in the Funding Statement section, we received no funding to conduct the research. We have modified the Acknowledgments section to avoid any confusion. The revised version now indicates: “The authors are grateful to the General Hospital of Mexico, for providing access to the patient medical records and hospital facilities to carry out this research. They also want to thank the Program in Medical Sciences, Dentistry and Health of Master and Doctorate of UNAM and CONACYT, for helping YMG to conduct this research study. Page 23, lines 354-357. (clean file)

We have also removed any funding-related text from the manuscript.

3. Please include your amended statements within your cover letter; we will change the online submission form on your behalf.

**Response:** As requested, we have included our amended statements in our response to the reviewers’ comments.

4. Please note that in order to use the direct billing option the corresponding author must be affiliated with the chosen institute. Please either amend your manuscript to change the affiliation or corresponding author or email us at [plosone@plos.org](mailto:plosone@plos.org) with a request to remove this option.

**Response:** The corresponding author, Dr. Yvonne N. Flores, ([ynflores@ucla.edu](mailto:ynflores@ucla.edu)) is affiliated with the University of California, Los Angeles, which will cover the publication costs of this manuscript. This information is indicated on the title page of the manuscript, affiliations #7 and #8. Page 2, lines 22-26. (clean file)

5. Please review your reference list to ensure that it is complete and correct. If you have cited papers that have been retracted, please include the rationale for doing so in the manuscript text or remove these references and replace them with relevant current references. Any changes to the reference list should be mentioned in the rebuttal letter that accompanies your revised manuscript. If you need to cite a retracted article, indicate the article’s retracted status in the References list and also include a citation and full reference for the retraction notice.

**Response:** As requested, we have reviewed and modified the references so they are complete and correct. We do not cite any retracted papers.

6. Decision analysis and cost-effectiveness analysis are systematic approaches used to support decision-making under conditions of uncertainty that involve important trade-offs. These mathematical tools can provide patients, physicians, and policy makers with a useful approach to complex medical decision making. Your work is very interesting so I would like you conclusion highlight it as you recommend in line 314.

**Response:** As requested by the reviewer, we have included the following information to the Conclusions section: “The results of this research may help reduce post-operative hospital stays, which could lower the risk of in-hospital infections and associated deaths. In addition, a shorter hospital stay accelerates the process of reintegration into daily life, which contributes to improving the quality of life of patients. The results of this study may also be informative to policy makers who seek to improve the effectiveness, efficiency and quality of surgical services. Finally, we wish to highlight the relevance of our study in terms of its contribution to scientific knowledge, especially since the available evidence on the effectiveness of ARISCAT and spirometry is scarce.” Page 22, lines 341-348. (clean file)

7. In line 140 I suggest you specificate the model as mathematical model if it possible.

**Response:** We appreciate the suggestion and now indicate that we used a “deterministic mathematical model” Page 9, line 160. (clean file)

8. line 162 specificate the triangular distribution for estimating probabilities

**Response:** As requested by the reviewer, we now indicate that the triangular distribution was used to estimate the probabilities of the post-operative complications and mortality. We agree that this change will improve the reader's understanding. Page 11, lines 189-190. (clean file)

Reviewer #1
1-Consider expanding the description of the statistical techniques used.

**Response:** We appreciate the suggestion to expand the description of the statistical techniques we used, and have included the following information in the Methods section:

“The ACER was calculated by dividing costs by effectiveness and the ICER was calculated as (Costs1-Costs2) / (Effectiveness1 - Effectiveness2). Post-operative complications in the spirometry group were calculated as follows for the ACER and ICER respectively:

$POC ACER without spirometry\mathbf{=}\frac{Cost according to ARISCAT level without spirometry}{Decrease in POC without spirometry}$ vs

$$POC ICER without spirometry=\frac{Cost according to ARISCAT level without spirometry\mathbf{-}with spirometry}{Decrease in POC without spirometry\mathbf{-} with spirometry}$$

These same formulas were applied to the survival results, for each group with and without spirometry, and for each ARISCAT risk level.” Pages 10-11, lines 179-187. (clean file)

2-Consider expanding the data with criteria of complications and mortality.

**Response:** We appreciate the suggestion, and have included more information about the criteria used for the post-operative complications and mortality. The following text was added to the Methods section: “The criteria data for post-operative pulmonary complications was defined according to the same criteria used by the PERISCOPE study (atelectasis, bronchospasm, pleural effusion, pneumonia, respiratory failure, pneumothorax, and pulmonary embolism) [10]. Other postoperative complications included, surgical (hypovolemic shock, sepsis, abdominal pain, fistula, bleeding, paralytic ileus, vascular injury, perforation); metabolic (glycemic dysregulation, hepatic or renal failure); cardiovascular (cardiogenic shock and acute myocardial infarction); neurological (acute vascular events); and vascular (deep vein thrombosis). Mortality was recorded if it was directly related to a complication from the surgery that was performed.” Pages 8-9, lines 140-148. (clean file)
